# Heat Transfer in a Micropolar Fluid over a Stretching Sheet with Newtonian Heating

**DOI:** 10.1371/journal.pone.0059393

**Published:** 2013-04-02

**Authors:** Muhammad Qasim, Ilyas Khan, Sharidan Shafie

**Affiliations:** 1 Department of Mathematical Sciences, Faculty of Science, Universiti Teknologi Malaysia, Skudai, Malaysia; 2 Department of Mathematics, COMSATS Institute of Information Technology, Chak Shahzad, Islamabad, Pakistan; King’s College London, United Kingdom

## Abstract

This article looks at the steady flow of Micropolar fluid over a stretching surface with heat transfer in the presence of Newtonian heating. The relevant partial differential equations have been reduced to ordinary differential equations. The reduced ordinary differential equation system has been numerically solved by Runge-Kutta-Fehlberg fourth-fifth order method. Influence of different involved parameters on dimensionless velocity, microrotation and temperature is examined. An excellent agreement is found between the present and previous limiting results.

## Introduction

Understanding the flow of non-Newtonian fluids is a problem of great interest of researchers and practical importance. There are several natural and industrial applications of such fluids, for instance volcanic lava, molten polymers, drilling mud, oils, certain paints, poly crystal melts, fluid suspensions, cosmetic and food products and many others. The flow dynamics of non-Newtonian fluids can be described by non-linear relationships between the shear stress and shear rate. Further these fluids have shear dependent viscosity. In literature there exist many mathematical models with different constitutive equations involving different set of empirical parameters. The micropolar fluid model is adequate for exocitic lubricants, animal blood, liquid crystals with rigid molecules, certain biological fluids and colloidal or suspensions solutions. The micromotion of fluid elements, spin inertia and the effects of the couple stresses are very important in micropolar fluids [1], [2]. The fluid motion of the micropolar fluid is characterized by the concentration laws of mass, momentum and constitutive relationships describing the effect of couple stress, spin-inertia and micromotion. Hence the flow equation of micropolar fluid involves a micro -rotation vector in addition to classical velocity vector. In micropolar fluids, rigid particles in a small volume element can rotate about the centroid of the volume element. The micropolar fluids in fact can predict behavior at microscale and rotation is independently explained by a microrotation vector. More interesting aspects of the theory and application of micropolar fluids can be found in the books of Eringen [3] and Lukazewicz [4] and in some studies of Peddieson and McNitt [5] Willson [6] Siddheshwar and Pranesh [7], [8], Siddheshwar and Manjunath [9].

During the past few decades, several researchers have concentrated on the boundary layer flows over a continuously stretching surface. This is because of their in several processes including thermal and moisture treatments of materials in metallurgy, in the manufacture of glass sheets, in textile industries in polymer processing of chemical engineering plants. Further the stretching flow with heat transfer is quite important in polymer extrusion, cable coating etc. The boundary layer flow of a viscous fluid over a stretching sheet was initially studied by Crane [10], then followed by many investigators for the effect of heat transfer, rotation, MHD, suction/injection, non-Newtonian fluids, chemical reaction etc. It is well known that in many industrial processes, heat transfer is an integral part of the flow mechanism. Now there is an abundant literature available on the flow induced by a stretching sheet with heat transfer [11]–[20]. Heat transfer characteristics are dependent on the thermal boundary conditions. In general, there are four common heating processes representing the wall-to-ambient temperature distribution, prescribed surface heat flux distribution, and conjugate conditions, where heat transfer through a bounding surface of finite thickness and finite heat capacity is specified. The interface temperature is not known a priori but depends on the intrinsic properties of the system, namely, the thermal conductivities of the fluid and solid. In Newtonian heating, the rate of heat transfer from the bouncing surface with a finite heating capacity is proportional to the local temperature surface which is usually termed as conjugate convective flow (see Merkin [21], Lesnic et al. [22], Chaudhary and Jain [23], Salleh et al. [24], Makinde [25]). Salleh et al. [24] numerically investigated the boundary layer flow of viscous fluid over a stretched surface in the regime of Newtonian heating. Numerical solution of the differential system is obtained by Keller box method. Desseaux and Kelson [26] investigated the flow of a micropolar fluid over a stretching sheet. In another attempt, Kelson and Desseaux [27] have investigated the effects of surface conditions on the flow of a micropolar fluid over a stretching sheet. The presented the closed form solution using the perturbation method and made a comparison between the analytical solution with numerical solution obtained by shooting method with fourth-order-Runge-Kutta algorithm. Bhargava et al. [28] studied mixed convection flow of a Micropolar fluid over a porous stretching sheet by implementing finite element method. The stagnation point flow of a micropolar fluid over a stretching surface has been discussed by Nazar et al. [29]. The steady MHD mixed convection flow towards a vertical stretching surface immersed in an incompressible micropolar fluid was investigated by Ishak et al. [30].

To the best of authors’ knowledge, the flow of micropolar fluid over a stretching sheet with heat transfer in the presence of Newtonian heating has not been addressed so far. The resulting problems are solved numerically and solutions obtained are compared with the existing results. It is found that the present results are in a very good agreement. Variations of several pertinent physical parameters are also analyzed in detail by plotting graphs.

### Basic Equations

We consider the steady boundary layer flow of an incompressible Micropolar fluid induced by a stretching surface. The sheet is stretched with a velocity 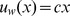 (where 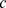 is a real number). The heat transfer in the presence of Newtonian heating is considered. The governing equations of the boundary layer flow in the present study are
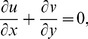
(1)

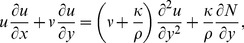
(2)

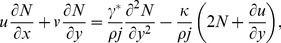
(3)

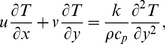
(4)where 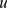 and 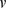 are the velocity components parallel to the 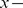 and 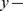
axes, respectively, 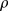 the fluid density, 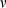 the kinematic viscosity, 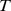 is temperature, 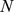 the microrotation or angular velocity, 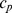 the specific heat, 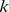 the thermal conductivity of the fluid, 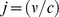 is microinertia per unit mass, 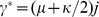 and 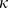 are the spin gradient viscosity and vortex viscosity, respectively. Here 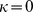 corresponds to situation of viscous fluid and the boundary parameter 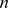 varies in the range 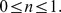 Here 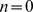 corresponds to the situation when microelements at the stretching sheet are unable to rotate and denotes weak concentrations of the microelements at sheet. The case 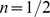 corresponds to the vanishing of anti-symmetric part of the stress tensor and it shows weak concentration of microelements and the case 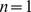 is for turbulent boundary layer flows.

The boundary conditions of the present problem are [24]

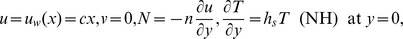


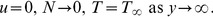
(5)


Here 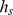 is heat transfer coefficient and 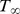 is the ambient temperature. In terms of similarity variables, we write
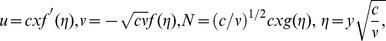


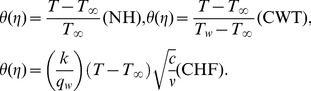
(6)


Introducing above Eqs. (6) into Eqs. (1)–(5) one has
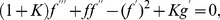
(7)

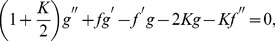
(8)

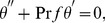
(9)

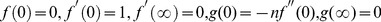
(10)

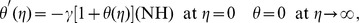


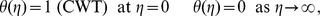


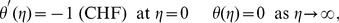
(11)where 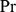 is the Prandtl number, 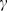 is the conjugate parameter for Newtonian heating and micropolar parameter 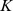. These quantities are given by


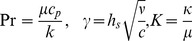
(12)

The skin friction coefficient 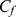 and local Nusselt number 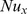 are
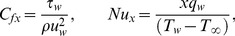
(13)in which the wall skin friction 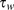 and the heat transfer 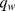 from the plate can be expressed as follows:



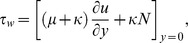


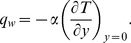
(14)


Now using Eqs. (6) and (14) into Eq. (13) we have
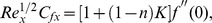


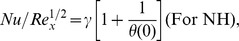


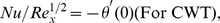


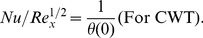
(15)where 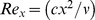 is the local Reynolds number.

## Results and Discussion

**Figure 1:**
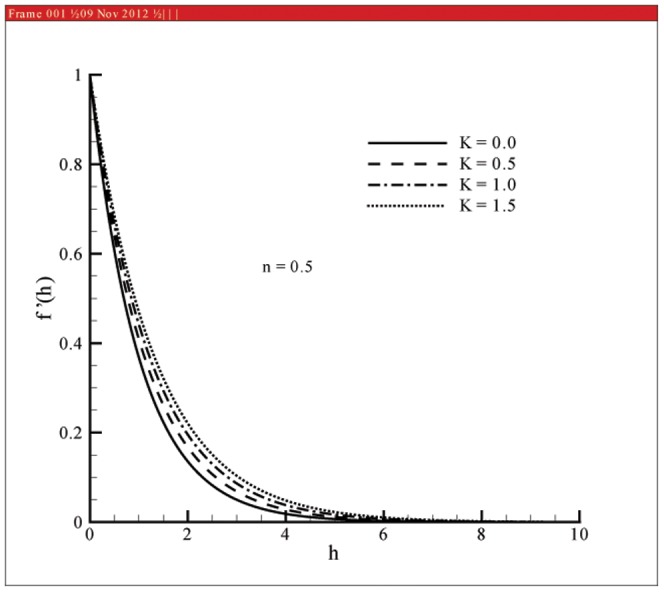
Influence of 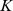 on velocity profile 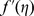 when 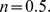

**Figure 2:**
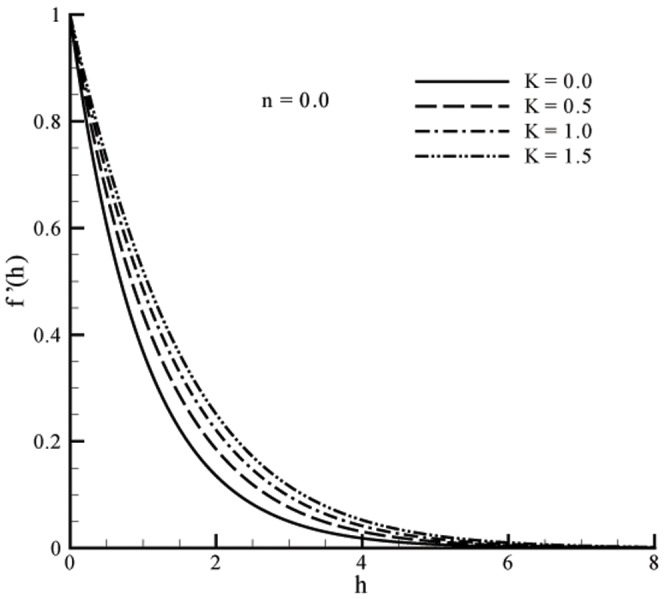
Influence of 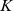 on velocity profile 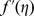 when 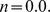

**Figure 3:**
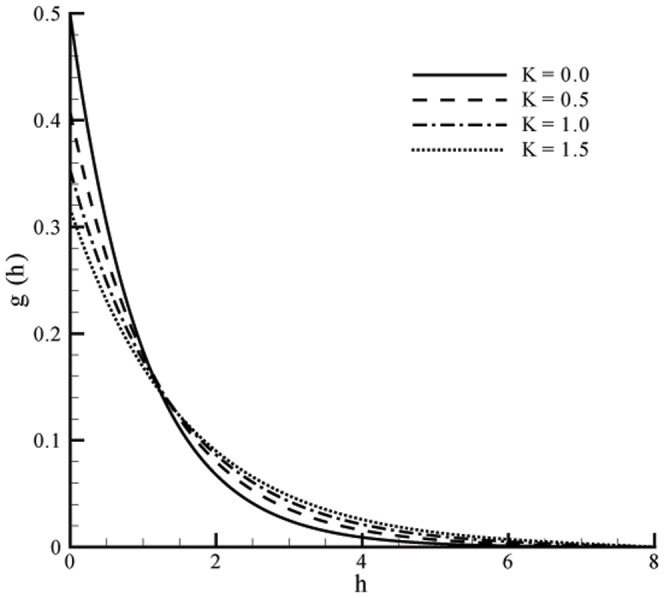
Influence of 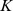 on microrotation profile 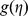 when 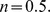

**Figure 4:**
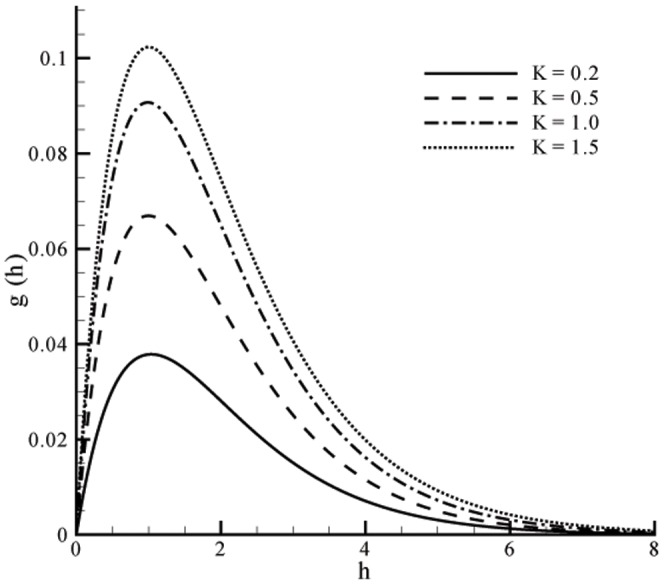
Influence of 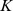 on microrotation profile 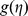 when 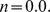

**Figure 5:**
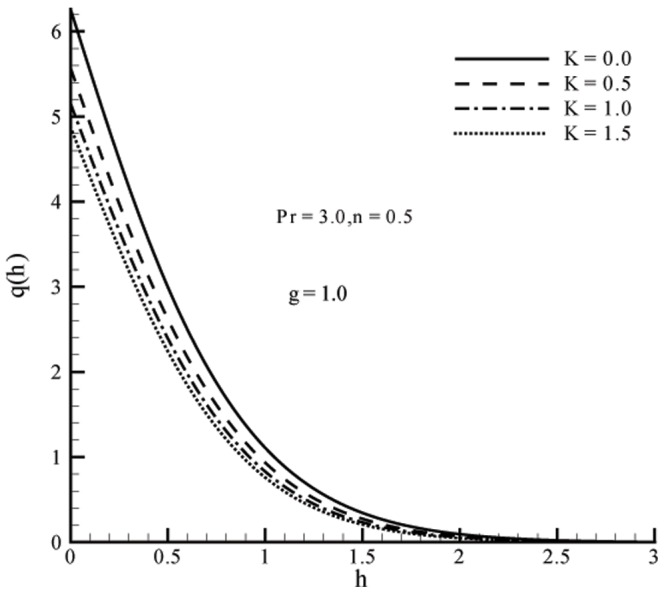
Influence of 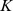 on temperature profile 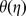 when 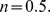

**Figure 6:**
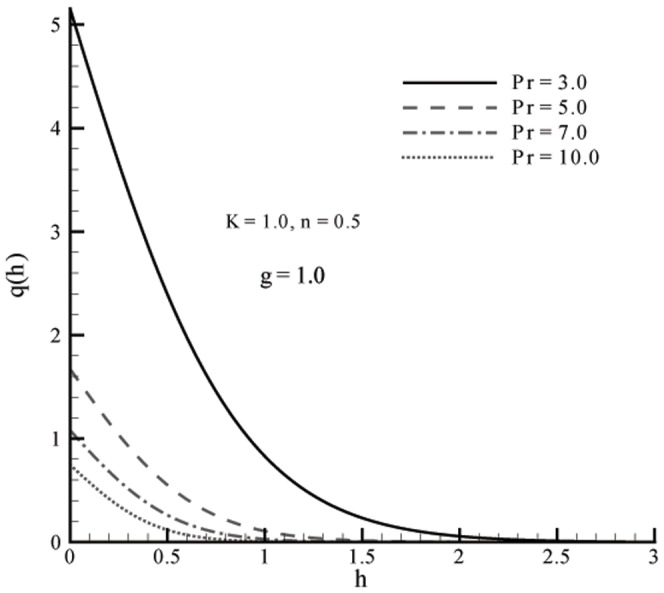
Influence of 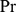 on temperature profile 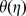 when 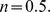

**Figure 7:**
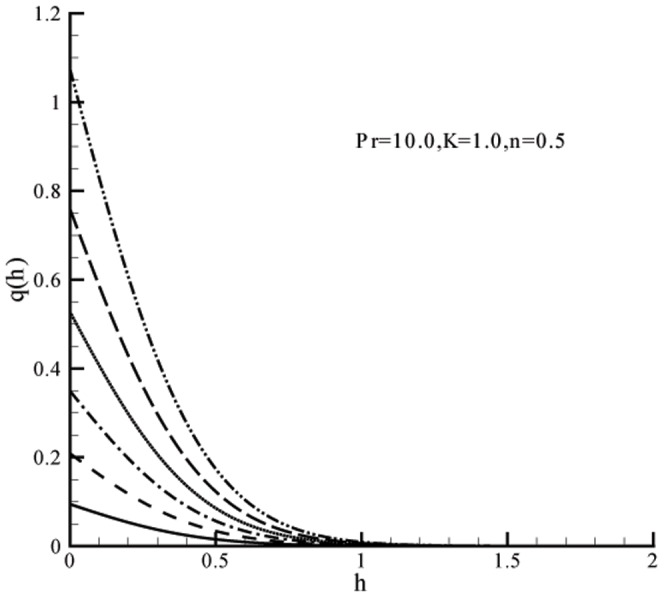
Influence of 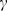 on temperature profile 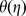 when 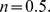

This section the effects of different parameters on the velocity, microrotation and temperature profiles (Fig. 1, 2, 3, 4, 5, and 6). Skin friction coefficient and the local Nusselt number are also Computed (see Tables 1, 2, 3, and 4). To authenticate our numerical solution a comparison is given in Tables 1, 2, 3, and 4 with already existing results in literature and both solutions are found in good harmony. The results for viscous fluid can be obtained when 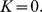 Figs. 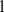 and 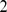 represent the velocity profiles for various values of vortex viscosity parameter 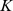 when 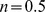 and 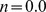 respectively. It is seen that results here are similar in both cases but change in Fig. 1 is slightly smaller when compared with Fig. 2. From Figs. 3 and 4 we can also observed that the microrotation profile for 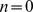 is different than 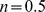. Fig. 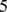 displays the effects of vortex viscosity parameter 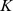 on temperature profiles 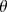. It is obvious that the increasing values of 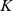 decreases temperature 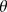. Fig. 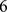 depicts the effects of conjugate parameter 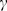 for Newtonian heating. For 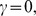 an insulated wall is present and constant surface temperature can be recovered when 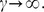 It is found that temperature increases with an increase in 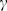. It is also noticed that the thickness of thermal boundary layer increases with an increase in 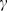. Further Eq. (9) has no meaningful solution for a very small Prandtl number, i.e. 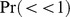. This is obvious in the sense that for 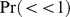 Eq. (9) reduces to 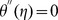 which has the solution 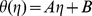 (where 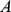 and 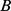 are the constants ). The boundary conditions are not satisfied by this 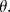 (see 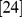) Fig. 7 examine the effects of Prandtl number on the temperature. An increase in Prandtl number 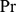 decrease the temperature 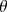. Note that 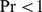 corresponds to the flows for which momentum diffusivity is less than the thermal diffusivity. An increase in the weaker thermal diffusivity therefore results in a thinner thermal boundary layer. The solution for Newtonian fluid 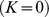 reduces to that derived by Salleh et al.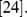 To authenticate our present numerical solution by Runge-Kutta-Fehlberg fourth-fifth order method with the exact solution and numerical solution obtained by Keller- box method a comparison is given in Tables 1, 2, 3, and 4 with already existing results in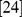. All the solutions are found in good harmony. In Table 1, values of local Nusselt number are compared with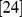 for the case of constant wall temperature (CWT). From this table we observed that the results obtained by Runge-Kutta-Fehlberg fourth-fifth order method are very closed to exact solution as compared to Keller-box method. From Table 2. it is found that the values of local Nusselt number are comparable with the results obtained by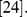 Table 3. presents the 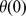 and 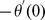 for various values of 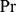 when 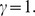 On comparison with Tables 1 and 2 for the cases of CHF and CWT, the trend for NH case is found similar to the CHF case but different from the CWT case. It is also observed that for Newtonian case both 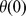 and 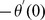 decreases as 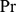 increases. From Table 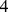 it is noticed that the magnitude of skin friction coefficient increases for large values of 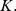


**Table 1:** Comparison of 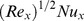 for different values of 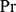 when 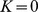 for CHF case.

Pr	*θ*(0) (CHF)		
	[24]		Present
0.72		2.15902	2.15916
1	1.71816	1.71828 1.71816	1.71828 1.71816
3	0.85819	0.85817	0.85819
5	0.63773	0.63770	0.63773
7	0.52759	0.52755	0.52758
10	0.43327	0.43322	0.43327
100	0.12877	0.12851	0.12877

**Table 2:** Comparison of 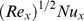 for different values of 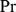 when 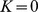 for CWT case.

Pr	−*θ'*(0)(CWT)		
	[24]		Present
0.72		0.46317	0.46360
1	0.58202	0.58198	0.58202
3	1.16525	1.16522	1.16525
5	1.56805	1.56806	1.56805
7	1.89540	1.89548	1.89542
10	2.30800	2.30821	2.30800
100	7.76565	7.76249	7.75826

**Table 3:** Comparison of 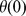 and 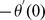 for different values of 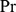 when 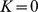 and 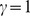 for NH case.

*θ*(0) (NH)	−*θ'*(0) (NH)			
Pr	[24]	Present	[24]	Present
3	6.02577	6.05168	7.02577	7.05168
5	1.76594	1.76039	2.76594	2.76039
7	1.13511	1.11682	2.13511	2.11682
10	0.76531	0.76452	1.76531	1.76452
100	0.16115	0.14781	1.16115	1.14780

**Table 4:** Values of Skin-friction coefficient 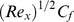 for different values of 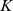 and *n*.

K\n	0.0	0.5
0.0	−1.000000	−1.000000
1.0	−1.367872	−1.224741
2.0	−1.621225	−1.414218
4.0	−2.004133	−1.732052

## Conclusions

The present study describes the boundary layer flow of Micropolar fluid with Newtonian heating. The main observations of this study are:

- Velocity and momentum boundary layer thickness are increasing functions of vortex viscosity parameter 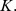

- Microrotation profile has a parabolic distribution when 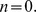

- The effect of vortex viscosity parameter 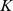 on velocity and temperature are quite opposite.
- Temperature and thermal boundary layer thickness are decreasing functions of vortex viscosity parameter 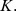

- An increase in the value of Prandtl number 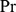 reduces the temperature and thermal boundary layer thickness.
- The present results in a limiting case 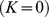 are found in excellent agreement with those of Salleh et al. [24].
- An appreciable increase in the magnitude of 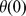 is shown for large values of 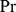 and 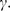

- The temperature profiles are also increased by increasing 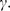
